# Ethnobotanical survey of trees in Fundong, Northwest Region, Cameroon

**DOI:** 10.1186/1746-4269-5-17

**Published:** 2009-06-25

**Authors:** Derek A Focho, Muh C Newu, Mendi G Anjah, Fongod A Nwana, Fonge B Ambo

**Affiliations:** 1 Department of Plant Biology, University of Dschang, P.O. Box 67, Dschang, Cameroon; 2 Department of Plant and Animal Sciences, University of Buea, Cameroon

## Abstract

Ethnobotanical investigations were conducted in Fundong Central Subdivision in the Northwest Region of Cameroon to identify trees growing in the area and collect information on their uses by the local people. This research covered a period of 12 months from May 2007 to April 2008. Ethnobotanical information was collected through the show-and-tell/semi-structured method and personal interviews during field trips. Three villages were investigated. A total of 82 tree species were identified belonging to 70 genera and 42 families. Among these species, 40 were widely used by the local people in traditional medicine to treat 48 human ailments. Tree species were also used for fuel wood, construction materials, wood carving and honey production. Leaves and barks were commonly used in traditional medicine while the wood, branches and the entire plants were commonly used for other purposes. In spite of the scarcity of natural forests in the study area, the local populations continue to depend on indigenous and exotic trees in their surroundings for their survival. There is therefore need for cultivation, protection and sustainable management of these valuable resources for rural livelihoods.

## Introduction

Ethnobotany, an area of human ecology, defines the interface between people and their forests, and offers clues needed for rural development based on sustainable yields of forest products [1]. The importance of timber and other tree products from outside forests is attracting increasing attention, to help meet growing demands and reduce pressure on natural forests and plantations [2]. Trees growing in open areas seem to have potentials to provide options for rural livelihoods and biodiversity conservation [3]. These trees can contribute to poverty mitigation serving as subsistence "safety nets" or low income "gap fillers". In addition to environmental stabilization, trees are useful for industrial, cultural, pharmaceutical, and socio-economic purposes to man, contributing billions of dollars yearly to the world's economy. Estimates have shown that about 90 percent of cooking and heating energy comes from trees [4]. Traditional societies in Africa and elsewhere have always used plants to promote healing and traditional medicine is still the predominant means of health care in developing countries [5-7].

The Fundong area (Boyo division) forms part of the Bamenda Highlands of Cameroon. Forest in this area is becoming so rare that it is possible to miss it entirely. Vegetation is currently dominated by grassland with patches of savannah and farms [8]. Trees growing in the open areas of this region can contribute to the wide-ranging needs of the rural people. These trees are currently used in the region for multiple purposes such as honey production, food, dye, fibre, fodder, medicines, fuel wood, building materials and production of kitchen utensils. Some of these trees have support roles for sustainable agriculture, livestock production, and hunting activities while others have cultural, religious or judicial functions. Most of the activities are major income generating. For example, collection and marketing of the wide range of non-timber products such as edible fruits, nuts, seeds and medicines [9]. The barks of some trees are used to produce ropes, straps and traditional oil containers while the woods of some are often valued for fuel wood and furniture [10].

While the knowledge on the usefulness of these plants remains high, poor methods of exploitation, agriculture and over-exploitation are putting most species under pressure of extinction. Ethnobotanical studies have reported useful plant species in Cameroon [11,12] and in the Bamenda highlands [13-15] but no ethnobotanical surveys of trees in the open areas of Fundong have been conducted.

The purpose of this investigation was therefore to document the uses of indigenous and cultivated species of trees growing in the open areas prior to their possible elimination through urbanization, deforestation and social development.

## Materials and methods

### Study area

**Figure 1:**
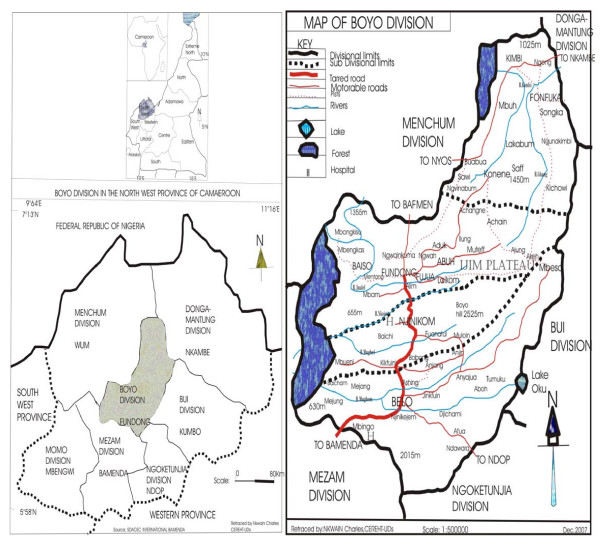
**Map of Boyo region**.

Boyo division is made up of four subdivisions (Fundong central, Bum, Belo and Njinikom). Fundong is the divisional headquarter and comprises several villages including Baiso, Abuh, and Fujua (Figure 1). This division falls between latitudes 6° 7' and 6° 24' N and between longitudes 10° 41' and 10° 31' E ([16], Fundong Rural council 2007). It shares territorial limits with five divisions of the Northwest Region of Cameroon (Menchum, Donga-Mantung, Bui, Ngoketunjia and Mezam divisions). The landscape is hilly with steep slopes increasing the rate of erosion. Deep valleys and flat plain-like features are limited to some depressions like Baiso. The name of the division originated from a hill (2220 m above sea level) situated at Njinikom called Boyo Hill. The division has a total surface area of 1592 km^2 ^with an estimated population of about 200000 inhabitants unevenly distributed across the entire surface area with Fundong alone having 47104 inhabitants. The rainy season starts from March and ends in November, with an average annual rainfall of 1200 mm. The dry season is from December to March, with February having the highest mean monthly temperature of 23°C. The vegetation of this area is afro-montane ranging from 500 m to 2230 m above sea level and is dominated by humid savannah with patches of sparse or thick montane forest galleries within depressions [10]. Farming is the main economic activity in the area with coffee, cola nuts, beans, corn and Irish potatoes being the main cash crops.

### Data collection and analysis

Field trips and collection of ethnobotanical data were carried out from May 2007 to April 2008.

Three villages were investigated in this study (Abuh, Fujua and Baiso) using the show-and-tell/semi-structured method adapted from [17]. Semi-structured questionnaires were used to interview the local population about their ethnobotanical knowledge of trees. Personal interviews and inquiries were also conducted during field trips. Interviewees were chosen without distinction of gender after seeking the consent from each respondent. People from all age groups, except children below 18 years were interviewed on their knowledge about the uses of trees in this region. The random sampling technique was used and a total of 110 questionnaires were distributed out to 70 males and 40 females in the site of the study. Information regarding the different uses of trees, parts used, origin, availability, and vernacular names was recorded. Informants were asked to name trees they knew, and to reveal the uses of the respective species. Informants often accompanied the investigators to the field to collect plant material. In cases of illiterate informants, photographs and fresh plant specimens from the field were presented to them and questionnaires were filled from their responses. Information was also recorded on the medicinal use of trees, plant parts used, diseases treated, modes of preparation and administration.

The working language was the dialect spoken in this region, Bikom, and the authors faced no language problems, one of them (Muh CN) being a native of the area. Due to the limited number of trees in the area, most people especially forestry workers knew each by name. Most plants were easily identified by their common or traditional names. Plants were initially identified by the authors and botanists from the Forestry Department, Fundong. Identifications were later validated in the Cameroon National Herbarium in Yaounde (YA). Collected specimens were preserved in the field using standard methods proposed by [18]. Voucher specimens were collected, preserved and deposited at the University of Dschang Teaching Herbarium.

Data on plant species, families, uses, origin, availability, and vernacular names and diseases treated were entered into excel worksheets where frequencies and abundance of each species were worked out. Data regarding plant uses were summarized as proposed by [19]. The frequency of occurrence of species was calculated in percentages per quadrat. Species present in 80–100% of quadrats were termed abundant, 60–70% was frequent, 20–40% was occasional or few and less than 20% were rare.

## Results

A total of 82 tree species were recorded in this study and all of them were reported as being useful in the lives of the local populations. Twenty five species did not have vernacular names amongst which 13 were exotic. Some of these species were introduced in the country during the colonial period as ornamental plants. They include *Callistemon viminalis, Eucalyptus globulus *and *Syzygium staudtii *from Australia, agroforestry species like *Calliandra callothyrsus*, *Leucaena leucocephala*, and *Casuarina equisefolia *from Central America, the *Citrus *species from Southeast Asia, *Cupressus benthami *and *Pinus sylvestris *from Europe and the *Podocarpus *species from Sao Tome Island. Most of the tree species are wild (64%) while 36% have been cultivated. A majority of respondents indicated that they use trees to supplement their monthly income and for nutritional purposes. This study revealed some common uses of trees in the site of the study amongst which medicinal use, construction materials, handicraft and fuel wood were the most important (Additional file 1). The most commonly used plant parts were wood, branches, barks and fruits. In some cases the entire plant was useful.

A total of forty species (49% of identified species) were used to treat 48 human ailments in the area of study. Treatments were administered topically, orally, by inhalation and as steam baths. The oral route was the most frequently used route of administration (74%) while inhalation was the least (4.6%). The leaves (42%) and stem barks (36%) were the most popular plant parts used in the various herbal preparations while roots, seeds and fruits were used occasionally. Flowers, nuts, latex and resins were rarely used. Decoctions, macerations and concoctions, necessitating a mixture of several plants are commonly used in treating malaria, infertility, typhoid, yellow fever, diarrhea, constipation, epilepsy, piles and sexually transmitted diseases (Additional file 1). Wounds, fractures, boils and other skin diseases are treated topically. The Bignoniaceae and Apocynaceae are the most represented families in terms of medicinal plant diversity. The most frequent plant species used include *Carica papaya*, *Prunus africana*, *Rauvolfia vomitoria*, *Kigelia africana*, *Spathodea campanulata *and *Psidium guajava. Prunus africana *is the most threatened medicinal plant in this area. The bark of *Bersama abyssinica *and seeds of *Carica papaya *are used as vermifuges (to repel intestinal worms) while the leaves of *Bridelia speciosa *are helpful in the treatment of diabetes. The roots and barks of *Rauvolfia vomitoria *are used throughout the area to calm mental patients.

## Discussion

Virtually all trees identified in the different families are useful in one way or the other in the lives of the rural population. Most species serve more than one function, for example in addition to its main function as fuel wood, *Bridelia speciosa *is widely used by the people in the manufacturing of tool handles (hoes, spears, axes, cutlasses and knives). Parts of this plant are also applied in traditional medical preparations for diabetes and constipation. Other species of the genus *Bridelia *have been reported elsewhere in Cameroon as important medicinal plants. For example, Focho et al. [7] report that the Aguambu-Bamumbu people use *B. micrantha *to treat cough and chest complaints. Adjanohoun et al [11] reported the use of young shoots of *B. atroviridis *to treat constipation. In addition to the use of *Eucalyptus globulus *as timber, this plant is the main source of fuel wood in the study regoin. Its leaves are also used in the preparation of remedies for cough and other diseases. *Ficus chlamydocarpa*, *F. elastica*, *Milletia courauri *and *Markhamia tomentosa *are other such multipurpose plants. Because of the relatively high population density of Fundong, land ownership disputes are common. Several *Ficus *species are used traditionally to demarcate boundaries. Fuelwood is an important comodity in the region and Fundong people cover great distances to collect it. Of the 82 species recorded 30 are used as fuelwood. The main species used are *Albizia gummifera*, *Schefflera manii, Nuxia congesta Gmelina arborea, Eucalyptus globulus *and *Pinus sylvestris*. Some like *Nuxia congesta *are becoming rare but are still collected for fuelwood. Bussmann et al. [20] have also reported that *Albizia gummifera *is an important source of firewood among the Maasai in Kenya. *Voacanga africana *is used in the area of study only for medicinal purposes. It is so much exploited for medicine that it has become a rare plant. Most of the trees recorded are considered to be few or rare. This is an indication of unsustainable methods of exploitation of these resources.

The application of leaves and stem barks in most herbal preparations can be attributed to the fact that these organs are known to accumulate in high concentrations, active components of most herbal preparations. These components which have been shown to relieve disease conditions in patients include alkaloids, tannins and inulin [21]. Leaves have also been reported to be the most commonly used plant part in other parts of Africa [22]. Trees are used to treat ailments ranging from common cold to complex pathological disorders relating to poor blood circulation, gastro-intestinal diseases, respiratory ailments, genital-urinary system as well as infertility, impotence, rheumatism and asthma. Some plants are used to treat more than one disease. For example, *Jatropha curcas *is used to treat epilepsy, gastritis, wounds, poisoning, mental disorders and as an abortifacient, *Spathodea campanulata *is used to treat malaria, mental disorders and hemorrhoids and *Kigelia africana *is effective in the treatment of male sexual impotence, rheumatism, pneumonia, wounds, filaria and cataract. The main methods of preparation of remedies were decoctions and concoctions while the mode of administration was oral for internal infections and topical for skin diseases.

Some of the species identified in this study have been reported to treat the same ailments elsewhere in Cameroon. Adjanohoun et al. [7] also reported the use of *Ficus exasperata *in the treatment of hemorrhoids in some parts of Cameroon. *Mangifera indica*, *Carica papaya*, *Citrus aurantifolia*, *Psidium guajava*, *Kigelia africana*, *Markhamia tomentosa *and *Spathodea campanulata *are used to treat ailments of the reproductive system in parts of West Africa [23,24]. Similar uses of these plants in the Bamenda Highlands have been documented by Tame and Thomas [25]. The study has revealed that medicinal plants still play a vital role in the primary health care of the people of Fundong.

Some individuals have created forests of *Eucalyptus *species for the production of electric poles and fuel wood. Others grow *Casuariana *and *Pinus *species extensively for Christmas trees and fuel wood. However, there are no state run plantations in the country for their cultivation.

## Conclusion

Many people in Fundong still depend on plants growing around them for most of their needs. The younger generations in this region are more interested in western lifestyles but some indigenous knowledge of plants still remains. Of the 82 tree species identified in the area of study, 40 are used to treat common ailments. All of the species are utilized by the local people to improve their livelihoods. The population has to be educated on propagation and conservation of the plants especially those used to treat the most common ailments.

## Competing interests

The authors declare that they have no competing interests.

## Authors' contributions

All the authors participated in the field work and in the preparation of the manuscript. DAF and MCN identified the plant specimens in the field before validation in the National Herbarium. All the authors participated in the analysis of data.

## Supplementary Material

Additional file 1 **Supplementary table**. Ethnobotany of trees in Fundong. Click here for file
