# Supplementary material for: Ethnobotanical survey of trees in Fundong, Northwest Region, Cameroon
Source: J Ethnobiol Ethnomed. 2009 Jun 25;5:17. doi: 10.1186/1746-4269-5-17 (PMC2708145; doi:10.1186/1746-4269-5-17)
Supplement: Additional file 1 — Supplementary table. Ethnobotany of trees in Fundong. [file 1746-4269-5-17-S1.pdf]

Table 1: Ethnobotany of trees in Fundong

No. spp	Family/ Species	Common/ local name	Habitat	Observation	Traditional uses	Freq	Parts used in medicine	Diseases treated	Preparation and Administration
1	**Agavaceae** *Dracaena arborea* Baker	Anchochom	IS	Available	Grown in compounds as a panacea for witchcraft. Wood carving, Construction of bridges, Musical instruments	8	Leaves<br><br>Whole plant	Liver disorders<br><br>Witchcraft	Decoction is taken orally.<br>Plant is grown in the compound.
2	**Anacardiaceae** *Mangifera indica* L.	Mango tree	ES	Available	Fruits are eaten, Medicine Agroforestry, Honey production,	26	Bark<br><br>Leaves	Dental caries<br><br>Syphilis, Malaria, Typhoid fever	Pulverized bark is applied topically or hot decoction is used as mouth wash.<br>Concoction with the bark of *Persea americana* is taken orally.
3	**Annonaceae** *Annona muricata* L.	Sweetsop	IS	Available	Used in agroforesty systems, Fruits are eaten	5			
4	**Annonaceae** *Annona squamosa* L.	Soursop	IS	Few	Fruits are eaten, Used in agroforesty	9			

					systems				
5	**Annonaceae** *Artocarpus altilis* (Pakinson) Fosberg	Bread fruit	IS	Few	Fruits are eaten, Provides timber and shade in farms, Medicine	3			
6	**Apocynaceae** *Alstonia boonei* De Wild	-	IS	Few	Fuelwood, Medicine	17	Roots<br>Bark<br>Latex	Epilepsy<br>Mental retardation, malaria, hernia<br>Snake bite	Decoction is taken orally.<br>Cold infusion is taken orally or as enema until healed.<br>Latex of stem is applied topically.
7	**Apocynaceae** *Rauvolfia vomitoria* Afzel.	Ijah	IS	Few	Medicine, Honey production	27	Root bark<br>Bark<br>Leaves	Snake bite<br>Hypertension,<br>Menstrual pains, Intestinal worms	Paste is applied topically.<br>Decoction is taken orally.<br>Pulverized with water is taken as an enema.
8	**Apocynaceae** *Voacanga africana* Stapf.	Utong	IS	Rare	Medicine	23	Bark<br>Fruits<br>Leaves	Poisons<br>Vision<br>Gonorrhoea	Bark decoction is taken orally.<br>Juice is used as eye drop.<br>Concoction with *Vitex doniana* fruits is taken

						Roots	Carious teeth	orally.<br>Pulverized root bark is applied topically.
9	**Araliaceae**<br>*Polyscias fulva* (Hiern) Harms	Umbrella tree	IS	Few	Wood carving, Medicine, Honey production, Musical instruments	17	Leaves<br><br>Bark	Jaundice, Headache<br>Pneumonia	Infusion is taken orally.<br>Concoction with P*ennisetum purpureum* is taken orally.
10	**Araliaceae**<br>*Schefflera abyssinica* Horst.Ex. A. Rich.	Uwoh	IS	Available	Honey production, Fuelwood, Construction	3			
11	**Araliaceae**<br>*Schefflera manii* (Hook.f.) Harms	Uwos	IS	Many	Construction, Honey production, Fuelwood	7			
12	**Arecaceae**<br>*Cocos nucifera* L.	Coconut palm	IS	Few	Fruits are eaten, Fuelwood, Agroforestry	7	Fruit<br><br>Root	Difficult lactation, Dandruff, Scabies.<br>Liver ailments	Fruits are eaten.<br><br>Decoction is taken orally.
13	**Arecaceae**<br>*Elaeis guineensis* Jack.Jack	Asomme	ES	Available	Fuelwood, Fruits are eaten, Agroforesty, Honey production,	15	Wine<br><br>Kernel oil	Difficult lactation<br>Anticonvulsant,	Fresh wine is drunk<br>Oil is applied on the body and is taken orally with

					Medicine		Nuts	Dysentery	salt.<br>Nuts are eaten with pulverized leaves of *Psidium guajava*
14	**Arecaceae**<br>*Phoenix dactylifera* L	Date palm	ES	Few	Ornamentals	12			
15	**Bignoniaceae**<br>*Kigelia africana* (Lam.) Benth.	Atem	ES	Few	Medicine, Honey production	31	Bark,<br>Fruits<br>Leaves	Male sexual impotence<br>Rheumatism, Pneumonia, Wounds, Filaria<br>Cataract	Decoction is taken orally.<br>Concoction with lime is taken orally.<br>Pulverized leaves are applied topically.<br>Juice from leaves is applied as an eye drop.
16	**Bignoniaceae**<br>*Markhamia lutea* (Benth) K. Schum ex Engl	Eyngweh atum	IS	Few	Tool handles and wood carving, Honey production, Shade	2			
17	**Bignoniaceae**<br>*Markhamia tomentosa* K. Schum ex Engl ex	Eyngueh	IS	Few	Tool handles and wood carving, Honey production	6	Bark	Male sexual impotence	Decoction is taken orally.

	Engl				Fuelwood, Planted on boundaries, Medicine				
18	**Bignoniaceae**<br>*Spathodea campanulata* P. Beauv	-	IS	Few	Fuelwood, Medicine	18	Bark<br>Leaves	Malaria<br>Mental disorders<br>Haemorrhoids	Decoction is taken orally.<br>Drop cold infusion into nostrils.<br>Pulverized leaves are applied topically
19	**Bixaceae**<br>*Bixa orellana* L.	Lipstick tree	IS	Few	Red pigment is used as a natural lipstick and in traditional ceremonies	5			
20	**Boraginaceae**<br>*Cordia millenii* Bak	-	IS	Available	Fuelwood, Mecicine	4	Leaves	Convulsion in children	Concoction with *Centella asiatica* is taken orally.
21	**Burseraceae**<br>*Canarium schweinfurthii* Engle	Ambah	IS	Few	Fruits are eaten, Honey production, Timber for consruction, Agroforestry.	15	Resin	Chest pain	Pulverized resin mixed with palm oil is taken orally.
22	**Burseraceae**<br>*Dacryodes edulis* (G. Don) H.J. Lam	Ajong	IS	Few	Fruits are eaten Agroforesty, Honey production.	18	Leaves	Convulsion in children	Concoction with *Centella asiatica* is taken orally.

23	**Caesalpiniaceae** *Cassia letptophylla* L	-	ES	Available	Ornamentals, Honey production, Fodder	16			
24	**Caricaceae** *Carica papaya* L.	Pawpaw	ES	Available	Fruits are eaten, Medicine, Honey production, Insecticides	34	Seeds, Fruits	Round worms, Diabetes, Anaemia, Asthma	Seeds are chewed and juice from ripe fruits is taken orally.
							Leaves	Malaria, Jaundice	Dry leaves are taken as tea. Concoction with *Cymbopogon citratuss* is taken orally
							Seeds	Dysentery	Seeds are chewed with salt.
25	**Casuarinaceae** *Casuarina equisefolia* Forssk.	-	ES	Few	Ornamental, Shade, Timber for constructing materials	7			
26	**Combretaceae** *Terminalia catapa* L.	Shade tree	IS	Few	Shade, Medicine	11	Leaves	Diabetes	Infusion or maceration is orally taken.
								Diarrhea	Pulverized leaves mixed with kernel oil are taken orally.
27	**Cupressaceae**	Cypress	ES	Available	Timber for	21			

	Cupressus benthamii Gord.				construction, Fuelwood, shade, Honey production, Insecticides.				
28	**Ericaceae** *Agauria salicifolia* Hook.f. Olive.	Ling	IS	Available	Fuelwood, ropes and construction material	11	Leaves, Bark	Syphilis	Decoction is taken orally
29	**Euphorbiaceae** *Aleurites montana* (Lour.) H.E.Wilson	Jung tree	IS	Rare	Ornamental, Shade	3			
30	**Euphorbiaceae** *Bridelia speciosa* Mull. Arg.	Sem	IS	Few	Tool handles, Fuelwood, Medicine	5	Leaves	Diabetes, constipation	Maceration or infusion of leaves is taken orally.
31	**Euphorbiaceae** *Croton macrostachyus* Hochst.ex Delile	Ejuam	IS	Many	Wood carving, Tool handles, Fuelwood, Insecticide, Honey production.	10	Bark	Pneumonia	Decoction is taken orally.
32	**Euphorbiaceae** *Jatropha curcas* L.	-	ES	Few	Medicine	9	Roots<br>Leaves<br>Seeds<br>Latex	Epilepsy<br>Gastritis<br>Abortifacient,<br>Mental disorders	Decoction is taken orally<br>Maceration is taken orally with lemon.<br>Seeds are burnt and eaten.<br>Pulverized dry seeds are taken orally

								Wounds<br><br>Poisoning	Latex is applied topically.<br>Pulverized seeds are eaten with food.
33	**Euphorbiaceae**<br>*Ricinodendron heudelotii* (Bails.) Heckel	Njangsang	IS	Few	Food additive, Timber	11			
34	**Euphorbiaceae**<br>*Sorindeia mibroedi* Engle & Brehmer	-	ES	Few	Ornamentals, Shade	3			
35	**Guttiferae**<br>*Garcinia kola* Heckel. Engle	Ibi-aku	ES	Few	Fruits are eaten, Timber, Medicine	9	Fruits<br><br>Root bark	Aphrodisiac, Gastritis<br>Tuberculosis<br>Asthma	Fruits are eaten.<br><br>Decoction is taken orally.
36	**Huaceae**<br>*Afrostirax lepidophyllus* Dougl.ex.Loud	Fulum	IS	Few	Food additive, Agroforestry	7			
37	**Ixoraceae**<br>*Ixora foliosa* Hiern.	Fegvu	IS	Many	Fuelwood, Construction materials	19			
38	**Lauraceae**<br>*Persea americana* Mil.	Pear tree	ES	Available	Medicine, Fruits are eaten Agroforestry, Seeds are used in dyeing activities	30	Leaves	Hypertention<br><br>Jaundice	Decoction with a bit of salt is taken orally<br>Decoction is taken orally
39	**Loganiaceae**	Fighia	IS	Rare	Musical	7			

	Nuxia congesta R.Br.ex.Fresen.				instruments, Honey production, Fuelwood				
40	**Meliaceae** *Azadirachta indica* L.	Neem	IS	Few	Medicine, Timber for construction	7	Leaves<br>Bark	Skin diseases<br>Malaria	Decoction or infusion is taken orally or as a bath.<br>Decoction is taken orally
41	**Meliaceae** *Carapa grandiflora* Sprague	Evin	ES	Few	Timber for construction, Fuelwood	11			
42	**Meliaceae** *Chlorophora excelsa* Benthand Hook f.	Iroko	ES	Few	Timber for construction, Medicine, Wood carving	3			
43	**Meliaceae** *Entandrophragma cylindricum* Sprague	Mahogany	IS	Few	Timber for construction, Fuelwood	14			
44	**Meliaceae** *Khaya senegalensis* (Desr.) A. Juss.	Mahogany	IS	Rare	Timber, Fuelwood	11			
45	**Melianthaceae** *Bersama abyssinica* Fresen	-	ES	few	Fuelwood, Medicine	2	Bark	Round worms	Decoction is taken orally
46	**Mimosaceae** *Acacia spectabilis* A.Cunn.ex.Benth.	-	ES	Available	Ornamentals, Improve soil fertility, Fodder	19			

47	**Mimosaceae**<br>*Albizia gummifera* (J.F.Gmel) C.A.	Fuwem	IS	Few	Fuelwood, Tool handles, Medicine	7	Bark ,<br>Leaves	Constipation, Bile<br>Filaria, eye pains<br>Abdominal pains	Decoction is taken orally<br>Decoction is taken orally and as eye drop.
48	**Mimosaceae**<br>*Calliandra callothyrsus* Meissn.	-	ES	Available	Ornamentals, Green manure, Fodder	28			
49	**Mimosaceae**<br>*Leucaena leucocephala* Lam. De Wit	-	ES	Available	Green manure, Agroforestry	29			
50	**Moraceae**<br>*Ficus chlamydocarpa* (Warb) ex Mildbr. &<br>Burret	Fig tree	IS	Rare	Fruits are used to feed domestic animals and birds, Planted on boundaries	17			
51	**Moraceae**<br>*Ficus elastica* Robx.	Male stick	IS	Available	Latex used to set traps for birds, Produces gum<br>Food for livestock<br>Planted on boundaries	13			
52	**Moraceae**<br>*Ficus exasperata* Vahl.	Sand-pepper tree	IS	Available	Sponge for cleaning household	7	Leaves	Haemorrhoids (Piles), Boils<br>Ringworms	Pulverized leaves are applied topically

					utencils, Medicine			Chest pain	Scrape affected part with leaf. Pulverized dried leaves are taken orally
53	**Moraceae** *Ficus natalensis* Hochst.	Fegvum	IS	Few	Timber for construction, Shade	3			
54	**Moraceae** *Ficus vogelii* (Miq.) Miq	Aloin	IS	Few	Fodder, Life fences	1			
55	**Myrsinaceae** *Maesa lanceolata* Forssk	Seim	ES	Rare	Fuelwood, Medicine	13	Fruits	Ascaries and round worms	Crushed fruits are orally taken as a single dose
56	**Myrtaceae** *Callistemon viminalis* L.	-	ES	Few	Ornamentals, Shade, Boundaries, Timber for construction.	12			
57	**Myrtaceae** *Eucalyptus globulus* (Labill.) Pers	Gum tree	ES	Available	Timber for construction, Fuelwood, Shade, Medicine	10	Flowers<br><br>Leaves	Cough Catarrh asthma<br>malaria, Jaundice	Cold infusion or decoction is taken orally<br>Decoction is taken orally
58	**Myrtaceae** *Psidium guajava* L.A	Futam	IS	Available	Fruits are eaten, Medicine, Tool handles	23	Leaves	Dysentery<br><br>Diarrhoea	Pulverized leaves are eaten with the nuts of *Elaeis guineensis.*<br>Maceration is

							Jaundice, Cough<br><br>Catarrh, Gonorrhoea	taken of young leaves is taken orally.<br>Concoction with *Prunus africana* is orally taken.
59	**Myrtaceae**<br>*Syzygium staudtii* (Engl.) Mildbr.	Ugweih	IS	Few	Honey production , Wood carving, Fuelwood	11		
60	**Papilionaceae**<br>*Entada abyssinica* Steud ex Rich	Felvung	IS	Rare	Medicine, Fuelwood	11	Bark	Gonorrhoea Syphilis, Candidiasis, Male and Female infertility	Decoction is taken orally.
61	**Papilionaceae**<br>*Erythrina excelsa* (Welw.) C.DC.	Sapelli	IS	Available	Ornamental, Shade and Construction	7			
62	**Papilionaceae**<br>*Millettia conrauri* De wild	Efume	IS	Few	Wood carving, Bark is used to make oil Containers and coffins for traditional leaders, Medicine	13			
63	**Papilionaceae**<br>*Newtonia*	Aloni	IS	Available	Wood carving,Tool	1			

	buchanannii Baker				handles, Fuelwood				
64	**Papilionaceae** *Pterocarpus soyauxii* Taub.	Cam wood	IS	Few	Red pigment is used in traditional marriages, Timber	21	Bark	Madness<br>Anaemia<br>Against evil spirits	Put pulverized bark into nostrils.<br>Decoction is taken orally<br>Powdered bark is taken orally
65	**Pinaceae** *Pinus sylvestris* Dougl.ex Loud	Pine	ES	Few	Timber, Shade, Fuelwood	9			
66	**Podocarpaceae** *Podocarpus mannii* Thunb.	Nkeng	IS	Many	Timber, Ornamentals, Fuelwood	16			
67	**Podocarpaceae** *Podocarpus latifolius* (Thunb) Mirb.	Nkeng–aku	IS	Available	Timber, Ornamentals, Fuelwood	3			
68	**Proteaceae** *Grevillea robusta* A.Cunn.	-	ES	Available	Honey production, Timber for construction, Shade	21			
69	**Rhamnaceae** *Maesopsis eminii* Engl.	Fang kijem	ES	Available	Timber, Shade, Fuelwood, Medicine	17			
70	**Rosaceae**	Kanda stick	ES	Many	Fuelwood,	29	Leaves	Hypertention	Decoction with a

	Prunus africana (Hook.F.) Kalkman				Timber, Medicine			Jaundice	bit of salt is taken orally. Decoction is taken orally.
71	**Rutaceae** *Citrus aurantifolia* Swingle	Lime	ES	Few	Fruits are eaten, Agroforestry plant	5	Leaves<br>Fruits	Abdominal pains<br>Filaria	Decoction is taken orally.<br>Juice is applied on the body after a bath.
72	**Rutaceae** *Citrus limon* Meyer	Grape	ES	Few	Fruits are eaten, Agroforestry	5	Fruits<br>Leaves	Cough, Loss of appetite<br>Goitre, impotence	Juice from ripe fruits is taken orally.<br>Maceration of leaves is taken orally.
73	**Rutaceae** *Citrus paradisi* Gros Sujet.	Lemon	ES	Available	Fruits are eaten, Agroforestry	14	Fruit	Tuberculosis	Juice is taken orally.
74	**Rutaceae** *Citrus sinensis* L.	Orange	ES	Available	Fruits are eaten, Agroforestry	10	Fruit<br>Leaves	Tuberculosis<br>Malaria	Juice is taken orally<br>Concoction with *Bidens pilosa* and *Carica papaya* is taken orally.
75	**Sapindaceae** *Allophylus bullatus* Radlk	-	IS	Few	Ornamental, Timber for construction	8			
76	**Solanaceae** *Cyphomandra betacea* (Cav)	Ajuanjongna	IS	Few	Fruits are eaten	9			

	Sendt.								
77	**Solanaceae** *Solanum torvum* L.	Finyiah	IS	Available	Grown in farms as a panacea for witchcrafts, Medicine	13	Fruit, bark<br>Leaves<br>Fruits, roots	Infertility<br>Gastritis<br>Pneumonia	Decoction is taken orally.<br>Leaves are chewed with limestone.<br>Pulverized roots and fruits in water are taken orally.
78	**Sterculiaceae** *Cola acuminata* (P.Beauv.) Scohott and Engl.	Ibi (Cola nut)	IS	Available	Fruits are eaten, Agroforestry, Medicine, Seeds used to dye traditional equipment	33	Bark<br>Fruits	Gonorrhoea<br>Cough	Decoction is taken orally.<br>Fruits are eaten.
79	**Thymeleaeceae** *Gnidia glauca* (Fresen.) Grilg.	Ejeo	IS	Available	Fuelwood, Construction materials	11			
80	**Ulmaceae** *Trema guineensis* (L.) Blume.	Fang kom	IS	Few	Toolhandles, Fuelwood, Medicine	21	Leaves	Male and female infertility	Maceration or infusion is taken orally
81	**Verbenaceae** *Gmelina arborea* L.	Abiuy	ES	Few	Timber for construction, Fuelwood, Tool handles	7			
82	**Verbenaceae** *Vitex doniana* Schum. & Thonn.	-	IS	Few	Timber, Fuelwood, Medicine	7	Bark	Syphilis	Concoction with bark of *Voacanga africana* is taken orally

Habitat: ES = Exotic species, IN = Indigenous species, IS = Introduced species.

No. spp: number of species Freq: frequency of respondents
